# Cumulative Exposure to Lead in Relation to Cognitive Function in Older Women

**DOI:** 10.1289/ehp.11846

**Published:** 2008-12-11

**Authors:** Jennifer Weuve, Susan A. Korrick, Marc A. Weisskopf, Louise M. Ryan, Joel Schwartz, Huiling Nie, Francine Grodstein, Howard Hu

**Affiliations:** 1 Department of Environmental Health, Harvard School of Public Health, Boston, Massachusetts, USA;; 2 Rush Institute for Healthy Aging, Rush University Medical Center, Chicago, Illinois, USA;; 3 Channing Laboratory, Department of Medicine, Brigham and Women’s Hospital, Harvard Medical School, Boston, Massachusetts, USA;; 4 Department of Epidemiology and; 5 Department of Biostatistics, Harvard School of Public Health, Boston, Massachusetts, USA;; 6 Department of Environmental Health Sciences, University of Michigan School of Public Health, Ann Arbor, Michigan, USA

**Keywords:** aging, blood lead, cognitive function, epidemiology, KXRF bone lead, women

## Abstract

**Background:**

Recent data indicate that chronic low-level exposure to lead is associated with accelerated declines in cognition in older age, but this has not been examined in women.

**Objective:**

We examined biomarkers of lead exposure in relation to performance on a battery of cognitive tests among older women.

**Methods:**

Patella and tibia bone lead—measures of cumulative exposure over many years—and blood lead, a measure of recent exposure, were assessed in 587 women 47–74 years of age. We assessed their cognitive function 5 years later using validated telephone interviews.

**Results:**

Mean ± SD lead levels in tibia, patella, and blood were 10.5 ± 9.7 μg/g bone, 12.6 ± 11.6 μg/g bone, and 2.9 ± 1.9 μg/dL, respectively, consistent with community-level exposures. In multivariable-adjusted analyses of all cognitive tests combined, levels of all three lead biomarkers were associated with worse cognitive performance. The association between bone lead and letter fluency score differed dramatically from the other bone lead-cognitive score associations, and exclusion of this particular score from the combined analyses strengthened the associations between bone lead and cognitive performance. Results were statistically significant only for tibia lead: one SD increase in tibia lead corresponded to a 0.051-unit lower standardized summary cognitive score (95% confidence interval: −0.099 to −0.003; *p* = 0.04), similar to the difference in cognitive scores we observed between women who were 3 years apart in age.

**Conclusions:**

These findings suggest that cumulative exposure to lead, even at low levels experienced in community settings, may have adverse consequences for women’s cognition in older age.

Impaired cognition and cognitive decline in older age are associated with heightened risks of subsequent physical disability (Greiner et al. 1996; McGuire et al. 2006; Raji et al. 2004) and hospitalization (Chodosh et al. 2004), even after control for general health status. Moreover, small decrements in cognition are strong predictors of eventual development of dementia (Blacker et al. 2007; Elias et al. 2000; Kawas et al. 2003; Linn et al. 1995; Morris et al. 2001; Small et al. 2000). For example, in studies of nondemented older adults, those who eventually developed cognitive impairment or dementia had average cognitive test scores at baseline that were only 0.15–0.9 standard units below the mean scores among individuals who remained cognitively intact (Linn et al. 1995; Morris et al. 2001; Small et al. 2000). The early, pre-clinical stages of disease represented by small cognitive decrements may be most amenable to intervention. The identification of modifiable risk factors for cognitive decline may provide important clues for delaying or even preventing dementia.

These modifiable risk factors potentially include exposures to environmental toxicants, and among the most historically pervasive and well-established neurotoxic pollutants is lead. Lead has been shown to be neurotoxic at progressively lower doses in children (Koller et al. 2004) and at high doses in occupationally exposed adults (Fiedler et al. 2003; Schwartz and Stewart 2000; Shih et al. 2007). Because lead is difficult to excrete (Agency for Toxic Substances and Disease Registry 2006), older adults, by virtue of their longer life spans, generally have accrued higher lead exposures than younger adults, whether these exposures originated from occupational or, more commonly, nonoccupational sources (Vig and Hu 2000). Comparatively little research has evaluated the relation of cumulative lead exposure to cognitive function and decline in older adulthood, although a growing body of research on this topic has emerged over the past decade. These studies generally have found inverse associations between indices of lead exposure and both cognitive function and change in cognitive function (Shih et al. 2007). However, women are underrepresented in this research. For example, in their review of studies investigating cumulative lead exposure and cognitive outcomes in adults, Shih et al. (2007) identified 21 studies, and in 16 of these studies, over 80% of participants were men. None of the studies reported results specific to women.

The measure of lead exposure is a critical feature of any study that examines lead exposure and cognition among older, community-exposed women in the United States Blood lead level is a gauge of recent lead dose, in contrast to concentration of lead in bone, which is an integrative measure of lead exposure over many years, in addition to being an endogenous source of lead (Hu et al. 2007). Thus, blood lead levels are likely to be less informative than bone lead about cumulative exposures in an environment characterized by low lead content in the present but relatively high content in the past, such as that in the United States after the phase-out of leaded gasoline in the 1980s.

Therefore, to better characterize the effects of recent and cumulative lead exposure on cognition in older women, we conducted a prospective study of both bone and blood lead levels in relation to cognitive function in a cohort of older, community-exposed women, hypothesizing that measures of lead exposure would be related to worse performance on the cognitive tests, but that associations would be stronger for bone lead levels, measures of cumulative exposure.

## Materials and Methods

### Study population

The Nurses’ Health Study (NHS) began in 1976 when 121,700 registered nurses, 30–55 years of age and living in 11 U.S. states, returned a questionnaire on their medical history and health-related behaviors (Colditz et al. 1997). Since then, the women have completed mailed questionnaires every 2 years. To date, the study has maintained follow-up of over 90% of the original participants.

Our study population came from two sub-samples of the NHS cohort that had previously been evaluated for lead exposure. The first was a sample of women participating in a case–control study of lead exposure and hypertension (Korrick et al. 1999). We invited women to take part in this study if they lived in the greater Boston, Massachusetts, metro politan area; did not have a history of major, chronic disease; and were not obese (body mass index ≥ 29 kg/m^2^). Women who remained free of major, chronic disease from 1990 to 1994 were invited to participate as controls, and women who first reported a diagnosis of hypertension between 1990 and 1994 were invited to participate as cases. Controls were frequency matched to cases by 5-year age groups. In total, between 1993 and 1995, 301 NHS participants agreed to participate and attended our outpatient General Clinical Research Center (GCRC), where they underwent the study evaluation, including measurement of their lead exposure.

The women in the second sample were originally recruited for a cohort study of lead exposure and osteoporosis. Similar eligibility criteria used for controls in the hypertension study applied here, with participants being free of chronic diseases during the recruitment period from 2000 to 2004. In total, 320 NHS participants attended our outpatient GCRC for evaluation including lead exposure assessment. In both studies of lead exposure, we measured lead content in blood and in both cortical and trabecular bone.

Cognitive assessments occurred from 1995 to 2005. Of the 621 women who participated in the lead exposure studies, 6 had died and 3 were too ill to participate in a cognitive assessment. We were unable to contact 17. Of those remaining, 8 (1.3%) declined participation. Thus, 587 women had cognitive assessments. Our analyses of tibia and patella bone lead included, respectively, all (587) and nearly all (586) of these women; 581 women had valid blood lead measurements and were included in analyses of blood lead and cognitive function.

### Lead exposure assessment

Participants visited the outpatient GCRC of the Brigham and Women’s Hospital for measurement of lead content in their bone by K-X-ray fluorescence (KXRF), a noninvasive technique for measuring skeletal lead content that can distinguish among very low lead burdens (Burger et al. 1990). The KXRF instrument provides an unbiased estimate of bone lead levels normalized to bone mineral content and expressed as micrograms of lead per gram of bone mineral. The instrument also provides an estimate of the uncertainty for each measurement equivalent to the standard deviation of repeated measurements. Negative estimates of bone lead concentrations may occur for lead values close to zero. Use of all point estimates without imposition of a minimum detectable limit has been identified as the most appropriate method of using these data in epidemiologic studies (Kim et al. 1995).

Bone lead measurements were made at each woman’s mid tibial shaft and patella. These sites are targets for bone lead research because the tibia consists mainly of cortical bone, and the patella of trabecular bone. The half-life of lead in trabecular bone varies by age and previous exposure, but in a cohort of older men, it has been estimated to be 8 years, whereas the half-life of lead in cortical bone is on the order of decades (Kim et al. 1997).

When we began measuring the women’s bone lead, we used an instrument developed by ABIOMED (Danvers, MA). A technical description and validity specifications of this instrument have been published elsewhere (Aro et al. 2000; Burger et al. 1990; Hu et al. 1990). In 1999, we replaced our prototype ABIOMED instrument with an upgraded instrument designed to improve measurement precision, with changes in the cadmium radiation source, adjustments to the geometry of the measurement procedure, and upgrades in both the software and specific hardware components of the system (Aro et al. 1994). Intercalibration data from persons who were measured on both instruments demonstrated a linear relationship between the two measurements with a slope of 0.87. Using this correction factor, we are able to combine data from our prototype and upgraded KXRF machines (Nie et al. 2008). To reduce the impact of any additional scaling differences in these readings on our epidemiologic analyses, we included in all of our bone lead regression models a term for lead substudy source, which effectively adjusts for instrument, because women from the hypertension substudy were assessed on the ABIOMED instrument and women from the osteoporosis study were assessed on the upgraded instrument.

We collected samples of blood in trace-metal–free tubes (with EDTA) and analyzed them for whole blood lead using graphite furnace atomic absorption with Zeeman background correction (ESA Laboratories, Chelmsford, MA). The instrument was calibrated with National Institute of Standards and Technology (NIST) Standard Reference Material (SRM) 955a, lead in blood (NIST, Gaithersburg, MD), after every 20 samples. Ten percent of samples were run in duplicate; at least 10% of the samples were controls and 10% were blanks. In tests on reference samples from the Centers for Disease Control and Prevention (Atlanta, GA) precision (coefficient of variation) ranged from 8% for lead concentrations of 10–30 μg/dL to 1% for higher concentrations. Compared with an NIST target of 5.7 μg/dL, 24 measurements by this method gave a mean ± SD of 5.3 ± 1.23 μg/dL. Eighteen percent of women in our study had blood lead levels below the minimum detection limit of 1.0 μg/dL; we recoded these values to be 1 μg/dL divided by the square root of 2 (0.71 μg/dL).

### Cognitive function assessment

Cognitive testing occurred as part of several substudies, although overall methods were identical in all participants. Of the 587 women included in our analyses, 72 (12%) were tested as part of a large-scale study of cognition that began in 1995 of NHS participants ≥ 70 years of age; 14 (2%) were tested in 2002 and 2004 as part of a study of cognition in “younger” older women and a study of Parkinson disease; and the remaining 501 women were tested during 2004–2005 to assess those in the lead study who had not been evaluated as part of these other studies. On average, cognitive assessments took place 5 years (25th to 75th percentile, 2–9.8 years) after lead exposure assessments.

All cognitive testing was administered using validated telephone interviews conducted by trained nurses. When testing began in 1995, we administered only the Telephone Interview for Cognitive Status (TICS; *n* = 587) (Brandt et al. 1988), a test of global cognition that is modeled on the Mini-Mental State Examination (MMSE) and evaluates functions such as orientation, registration, and immediate verbal memory (Folstein et al. 1975). A score < 31 on the TICS indicates cognitive impairment. We gradually added other tests to the battery; thus the sample size differs slightly across the cognitive tests. These tests include delayed recall of the 10-word list (*n* = 569) from the TICS to assess delayed verbal memory; the East Boston Memory Test (EBMT; *n* = 582) to assess immediate and delayed paragraph recall (Albert et al. 1991; Scherr et al. 1988); category fluency (*n* = 582) in which participants were asked to name as many animals as they could in 1 min (Goodglass and Kaplan 1983); and the digit span backwards test (*n* = 566), to assess working memory and attention (Lezak 1995). The women tested during 2004–2005 and in one of the small studies were slightly younger than the women participating in the large-scale study of cognition; to better discriminate the cognitive abilities of these younger women, we added two tests to their assessment battery. For the alphabetizing span test (*n* = 508), another test of working memory and attention, women were asked to recall in alphabetical order an increasingly longer list of unordered words (Craik 1990). We also tested letter fluency (*n* = 511) by asking them to name as many words beginning with “f” as they could in 1 min (Lezak 1995). For analyses, we calculated a composite score of verbal memory (*n* = 568), a strong predictor of Alzheimer disease (AD) development (Linn et al. 1995; Tabert et al. 2006), by averaging the *z* scores of the immediate and delayed recall of both the EBMT and the TICS 10-word list for all women who took these tests.

Previously, we extensively evaluated the reliability and validity of our telephone assessment procedure. Interviewers were highly consistent with each other in their scoring; we recorded all interviewers as they administered the cognitive battery, and then each interview was evaluated by multiple interviewers. Correlations between interviewers’ score assignments on any given test exceeded 0.95. We also found high reliability of test performance among 35 women given the TICS twice 31 days apart (Pearson *r* = 0.7). In a validation study we conducted among 61 women from the Religious Orders Study (Bennett et al. 2002) of similar age and educational status to our participants, we found a correlation of 0.81 between overall performance on our telephone interview and overall performance on an in-person interview. As with performance on in-person assessments, performance on our telephone assessment strongly predicts dementia diagnosis. In our clinical validation study, over 3 years, the risk of dementia was 8–12 times higher for women with poor general cognition and 26 times higher for those with poor episodic memory on our telephone interview.

### Statistical analysis

We performed separate analyses for each of the three lead biomarkers. For ease in comparing results for the different cognitive tests, we used each cognitive test’s *z* score, computed from the means and SDs in our study population.

We examined performance on the individual test *z* scores and verbal memory score, using multiple linear regression to estimate the mean difference in standardized cognitive test score (*z* score) per SD increment in levels of each of the three lead biomarkers. To summarize the overall association of each lead biomarker with cognitive performance, we used generalized estimating equation (GEE) models, initially treating the eight *z* scores as correlated repeated measures of cognitive function (Lefkopoulou et al. 1989). If all cognitive test scores reflect the same underlying phenomenon, these GEE models provide effect estimates that are more precise than those from the models of the individual test scores. These models also are able to accommodate missing cognitive scores, such as when a participant was not administered a test in the battery. For each lead biomarker, we fit a GEE model that simultaneously regressed the eight cognitive test *z* scores on the biomarker and covariates, producing a summary estimate of the mean difference in standardized cognitive test score per SD increment in lead biomarker. We allowed the working matrix of correlations between cognitive scores to be unstructured. In model fitting, we assumed that the lead biomarker–cognitive score association was the same across all cognitive tests (common exposure effect assumption). We tested this assumption by fitting a GEE model, as described above, but which also included cross-product terms between the lead biomarker of interest and indicators for each cognitive test (except for an arbitrarily chosen referent, the EBMT-immediate recall). A generalized score test with 7 degrees of freedom corresponds to the cross-product terms combined. Large values of the generalized score test indicate substantial deviation from the common exposure effect assumption, meaning that at least one of the cognitive tests differs significantly from the others in its association with lead and suggesting reconsideration of the individual cognitive tests included in the model.

We adjusted all models for factors evaluated near the time of the lead exposure assessment that may confound the association between lead and cognition or, to improve analytical precision, were strongly associated with cognition in previous work. These factors included age at lead exposure assessment, age squared, education (registered nurse degree, bachelor’s degree, advanced graduate degree), husband’s education (high school diploma or less, college degree, advanced graduate degree; an additional measure of socioeconomic status), alcohol consumption (measured by food frequency questionnaire as none, up to 1 drink/week, 2–6 drinks/week, ≥ 1 drink/ day), smoking (current, past, never), regular pattern of physical activity (energy expenditures computed from responses on a leisure activity frequency questionnaire, and divided into tertiles), aspirin use (nonuser, 1 time/ month to 2 times/week, ≥ 3 days/week), ibuprofen use (nonuser, current user), vitamin E supplementation (yes, no), menopausal status (yes, no) and postmenopausal hormone use (never, past, current). We also included terms for age at cognitive assessment, lead substudy, and cognitive substudy. In separate models, we further adjusted for vascular and mental health factors that might be either confounders or intermediates in the causal pathway between lead exposure and cognitive function, including high blood pressure, antihypertensive medication use, poor mental health on the mental health scale of the Short Form-36, and antidepressant use. (We did not adjust for other factors such as coronary heart disease and diabetes, because very few women in this relatively healthy subsample reported having these conditions.)

We conducted all analyses using SAS version 9 (SAS Institute Inc., Cary, NC), using PROC GENMOD to fit the GEE models. We used *p* < 0.05 as the level of statistical significance.

This study was approved by the institutional review boards of the Brigham and Women’s Hospital, the Harvard School of Public Health, and the University of Michigan. Study participants gave their written consent to participate in the studies of lead exposure and gave their verbal consent to participate in the cognitive portions of the study at the time of their cognitive assessment.

## Results

At the time of their lead assessments, the women in our study were 47–74 years of age (mean, 61 years). Nearly all (99%) identified themselves as non-Hispanic white. Typical of a nonoccupationally exposed population, they had relatively low concentrations (mean ± SD) of lead in their tibia (10.5 ± 9.7 μg/g), patella (12.6 ± 11.6 μg/g), and blood (2.9 ± 1.9 μg/ dL). These levels were about half of levels observed among a cohort of slightly older men (mean age, 67 years) also living in the greater Boston area (Hu et al. 1996). Lead levels in the two bone sites were more strongly correlated with each other (Spearman *r* = 0.44) than with blood lead (*r* = 0.18 and 0.23 for patella and tibia lead, respectively). As observed previously (Korrick et al. 2002), lead biomarker levels were higher among women who were older, had less formal education, were current or former smokers, and consumed more alcohol (Table 1). Among these “young old” women, only 22 (4%) scored < 31 on the TICS, indicating potential cognitive impairment, and lead biomarker levels were markedly higher in these women, by 5.4 μg/g, 8.1 μg/g, and 0.9 μg/dL in tibia, patella, and blood, respectively.

**Figure 1:**
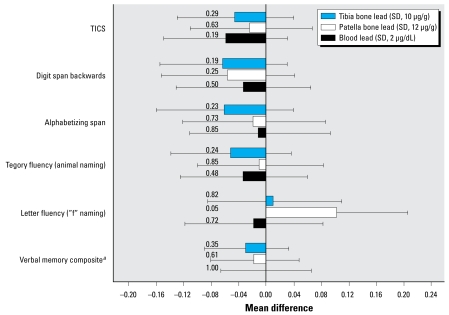
Adjusted mean difference (95% CI) in specific standardized cognitive test scores per SD increment in lead biomarker. Values are adjusted for age and age-squared at lead assessment, age at cognitive assessment, education, husband’s education, alcohol consumption, smoking status, physical activity, aspirin use, ibuprofen use, use of vitamin E supplements, menopausal status and postmenopausal hormone use, lead substudy source, and cognitive substudy source. Numbers left of bars indicate *p*-values. ***a***Average of *z*scores from the immediate and delayed recall of both the EBMT and the TICS 10-word list.

After adjusting for potential confounding factors, higher levels of the lead biomarkers were generally associated with worse performance on the individual cognitive tests, although none of these negative associations reached statistical significance (Figure 1). The inverse associations between lead exposure and cognitive function tended to be more pronounced for tibia lead than for the other two lead biomarkers. Of the negative associations for tibia lead, the strongest corresponded to the digit span backwards (*p* = 0.19) and the alphabetizing span tests (*p* = 0.23), both tests of attention and working memory.

Unexpectedly, higher levels of bone lead were associated with better performance on the letter fluency test, significantly so for patella lead (*p* = 0.05) (Figure 1). This discrepancy also appeared in results from GEE models in which we tested the common exposure effect assumption. These analyses revealed heterogeneity in the associations between bone lead and the cognitive tests, especially for patella lead, where the heterogeneity was statistically significant (*p* = 0.02). In particular, the association of patella lead with letter fluency test score was significantly different from the other patella lead–cognitive test associations (*p* = 0.006). Therefore, in addition to fitting GEE models that used all cognitive scores, we fit GEE models that excluded letter fluency scores.

Results from the GEE models that included all cognitive tests indicated that all three lead biomarkers were associated with worse overall cognitive function, although none of these associations was statistically significant (Table 2). However, when we excluded the letter fluency test from these models, these inverse associations became more pronounced, most markedly for patella bone lead. Notably, the association of tibia lead with overall cognitive function became statistically significant; for each SD increment in tibia bone lead concentration (10 μg/g), overall standardized cognitive scores were 0.051 units lower [95% confidence interval (CI), −0.099 to −0.003; *p* = 0.04]. To help interpret this finding, we contrasted the association of tibia lead with cognition to the association we found for age and cognition. Specifically, the 0.051-unit decrement in standardized cognitive score was equivalent to the difference in scores we observed between women in our study who were about 3 years apart in age. In these models, associations of both bone lead biomarkers with overall cognitive function were stronger than associations corresponding to blood lead.

Results remained unchanged when we further adjusted for potential vascular and mental health intermediates.

## Discussion

In this large study of healthy “young old” women, cumulative community-level exposure to lead, measured by concentration of lead in tibia bone, was associated with significantly worse overall performance on cognitive function tests. Specifically, the average decrement in cognitive test scores we observed for each SD increase in tibia lead corresponded to the decrement in scores we observed for each 3-year increase in age among women in our study.

Levels of two other lead biomarkers—patella lead and blood lead—were also associated with worse cognitive function, but these associations were not significant. This pattern of association suggests that lead exposures in the distant past may be more important than relatively recent exposures in influencing cognitive function in these women, because tibia lead levels measure cumulative exposures over the past decades, in contrast to the more recent exposures measured by patella and blood lead levels (Hu et al. 2007). Although tibia lead assessments cannot distinguish between chronic low-dose exposures and high exposures during a critical period in the past (Shih et al. 2007), chronic low-dose exposures likely prevailed among the women in this study, who probably incurred most of their exposures to lead from gasoline emissions and consumer products beginning in childhood and lasting at least through the 1980s, when these products were phased out in the United States.

The only large-scale study to report on lead’s association with cognition among older women occurred in the Study of Osteoporotic Fractures. Investigators cross-sectionally examined urban- and rural-dwelling women and found that higher blood lead levels predicted worse performance on several cognitive tests (Muldoon et al. 1996), although this association was present only among the rural-dwelling women. The reason for these restricted findings is unclear. One possibility, which is indirectly supported by our data, is that the measure of lead exposure—blood lead level—did not adequately capture the range of relevant exposures experienced by these U.S.-based women.

Numerous studies of adults with occupational exposures to lead have found adverse associations between current blood lead level and cognitive outcomes. Nonetheless, many of these studies also have found that measures of cumulative dose (e.g., serial blood lead measurements) generally are more strongly associated than current blood lead with adverse cognitive outcomes (Shih et al. 2007). These cohorts are characterized by high past and current exposures, with mean current blood lead levels often exceeding 25 μg/dL. In contrast, patterns of exposure among community- exposed adults in the United States reflect high past exposures followed by low current exposures, making current blood lead level a potentially less sensitive measure than cumulative exposure measures for evaluating the relation between lead exposure and cognitive aging in this population. This notion is supported by findings from studies of two community-based cohorts that suggest apparent effects of cumulative but not current low-level lead exposure on poor cognition and cognitive decline. In > 400 men (mean age, 67 years) participating in the Normative Aging Study, bone lead levels were associated with significantly steeper decline over a 3.5-year interval on the MMSE (Weisskopf et al. 2004) and three visuospatial tests (Weisskopf et al. 2007). Blood lead levels were cross-sectionally associated with performance only on a vocabulary test (Weisskopf et al. 2007) and the MMSE (Weuve et al. 2006). Similarly divergent findings for bone and blood lead initially emerged from a study of 994 older adults participating in the Baltimore Memory Study, in which tibia lead levels—but not blood lead levels—were associated with worse performance on all seven cognitive domains tested; however, these associations were substantially attenuated and no longer significant upon further adjustment for education, race/ethnicity and wealth (Shih et al. 2006), closer to the analytical framework of our study. A third study of 533 young adults (mean age, 24 years) found significant associations between living next to a lead smelter during childhood and performance on several cognitive tests (Stokes et al. 1998).

Few large-scale studies of cumulative lead exposure and cognition in older adults have included women, and none has reported results that are specific to women (Shih et al. 2007). The present study indicates that cumulative lead exposure may adversely affect cognitive aging even among women, whose exposure to lead is typically lower than that of men. This association has important consequences for public health, because impaired cognitive function is a strong risk factor for dementia (Bennett et al. 2002; Kawas et al. 2003; Morris et al. 2001) and because women have a higher lifetime risk than men of developing dementia (Andersen et al. 1999; Hebert et al. 2001; Ott et al. 1998; Seshadri et al. 1997).

More generally, although lead levels in the environment have fallen dramatically in the past two decades, many older adults have endured protracted exposures to lead in the preceding decades and have accumulated lead in their skeletons. Together with previous findings, our results have important implications for the cognitive functioning of this growing population of older adults. In the United States, the population of persons ≥ 65 years of age is projected to double between 2000 and 2030 (He et al. 2005), leading to a rapid rise in the number of individuals afflicted with age-related dementia. This phenomenon will likely be echoed throughout the globe (Ferri et al. 2005). One model has forecasted that a broadly applied intervention, such as a regulatory intervention that delays the onset of AD by 2 years, could reduce the number of prevalent cases in the United States by about 2 million over a 40-year interval (Brookmeyer et al. 1998). Thus, even if lead has a subtle effect in accelerating cognitive aging, given the pervasiveness of lead exposure in the United States and globally, widespread reductions in this exposure could have a substantial impact on the burden of cognitive impairment in the population.

Chronic, low-dose exposure to lead may adversely affect cognitive functioning in older age through several actions. Chiefly, lead can damage and eventually kill neurons through its oxidative toxicity, whereby lead both induces oxidative stress (Acharya and Acharya 1997; Adonaylo and Oteiza 1999b) and impedes responses to oxidative stress (Adonaylo and Oteiza 1999a; Ercal et al. 1996) in the brain. Oxidative stress, in turn, appears to be integral to the pathogenesis of cognitive decline and dementia (Andersen 2004; Markesbery and Lovell 2007). Lead also accumulates in neural mitochondria, where it eventually generates the abnormal release of calcium and induces apoptotic cell death (Anderson et al. 1996; Fox et al. 1997; He et al. 2000; Silbergeld 1992). Chronic exposure to lead is followed by astrogliosis in the hippocampus, indicating neuronal injury or death in a region that is critical for learning and memory function (Selvin-Testa et al. 1994). Acutely, lead also appears to interfere with calcium-dependent enzymes (Toscano and Guilarte 2005) as well as cholinergic, glutaminergic, and dopaminergic neuro-transmitter systems—all integral components of cognition (Cory-Slechta 1995).

Several limitations of our study warrant consideration. It is unlikely that our study findings directly reflect the acute cognitive effects of lead because current exposure levels, indicated by blood lead levels, were distributed over a very limited range. However, as a result of these limited current exposures, our study provides further evidence that past and cumulative exposures—apart from current exposures—may have chronic cognitive effects.

Previous studies have identified inverse associations between cumulative lead exposure and visuo spatial ability (Barth et al. 2002; Schwartz et al. 2000; Shih et al. 2006; Stewart et al. 1999; Weisskopf et al. 2007), but we were not able to explore this association in our study due to practical limitations in administering the cognitive battery by telephone. However, telephone testing has important advantages and enabled us to maximize recruitment into the cognitive study.

In addition, the GEE analysis was useful for summarizing our results and effectively optimized the precision of our effect estimates. Use of these models is contingent on reasonably homogeneous associations between lead and cognitive function for all cognitive tests included; however, the puzzling association of higher patella lead with better performance on the letter fluency test violated this assumption. This appears to be a unique finding, likely due to chance. Alternatively, this finding may hint that lead-induced cognitive impairments in older age overlap with those of AD. In AD, deficits in semantic fluency (e.g., category fluency) are common, whereas deficits in phonemic fluency (e.g., letter fluency) are not (Henry et al. 2004; Rascovsky et al. 2007). However, this explanation requires confirmation from future research.

Our single assessments of cognitive function do not directly capture change in cognitive function, nor do they evaluate dementia status. The women in our study were in their 50s and 60s at the time of their lead assessments, and were 5 years older, on average, when cognitive testing occurred. At these ages, dementia is still relatively rare (Rocca et al. 1998), but subtle decrements in cognition may be considered a preclinical stage of the condition, preceding it by many years (Elias et al. 2000; Kawas et al. 2003; Morris et al. 2001). Nonetheless, the direct evaluation of lead exposure in relation to cognitive change is of great interest, and repeated cognitive assessments will be conducted.

It is possible that our results were influenced by selection processes, although the direction of the ensuing bias, if any, is not altogether clear. About half of the participants were women from a case–control study of hypertension. Because lead exposure appears to be related to hypertension (Navas-Acien et al. 2007), it is possible that we overestimated lead’s adverse cognitive effects if cognition, too, was related to participation. Nonetheless, for our cognitive study, the cases from the hypertension study represented only 14% of participants. Conversely, the women in our study were healthy at enrollment, free of chronic diseases (except hypertension alone for cases); they also had low exposures to lead. Thus, again assuming that cognition was related to participation, then the overrepresentation of women who had both excellent cognition and low exposures to lead might have led us to underestimate lead’s adverse cognitive effects.

Finally, as in any observational study, our results could be confounded by unmeasured or mismeasured factors. The NHS cohort is fairly well-characterized, however, and is more homogeneous in terms of occupational and socioeconomic factors than most community-based cohorts. In addition, our results were robust to adjustment for numerous potential confounders, including education and husband’s education, indicators of socioeconomic status. It remains possible that the lead exposure during adulthood that we measured directly via tibia lead is a proxy for lead exposures and its consequences endured during childhood and, therefore, that the association between tibia lead and cognition mis specifies the importance of adult exposures. Although our data are insufficient to satisfactorily confirm or refute this possibility, we examined our findings in the presence and absence of adjustment for educational attainment—a blunt indicator of the consequences of childhood lead exposure—and the findings were unchanged.

In summary, in this study of 587 “young old” women who had community-level exposures to lead, higher levels of tibia lead (a measure of cumulative dose) were significantly associated with worse overall performance on a series of cognitive tests. In contrast, associations between a measure of recent lead exposure and cognition were weaker and not significant. This pattern of results suggests that, even in the absence of substantial current exposures to lead, chronic, low-level historical exposures to lead may have adverse consequences for the cognitive aging of women and thus merit further research.

## Figures and Tables

**Table 1:** Levels of lead exposure biomarkers (mean ± SD) by women’s characteristics.

		Lead biomarker^a^					
		Tibia		Patella		Blood	
Characteristic	No. (%)^b^	Lead (μg/g)	*p*-Value	Lead (μg/g)	*p*-Value	Lead (μg/dL)	*p*-Value
Age at lead exposure assessment (years)							
47–54	92 (16)	10.6 ± 8.3	0.0004	14.6 ± 11.0	0.001	2.6 ± 2.0	0.4
55–59	133 (23)	9.0 ± 8.7		10.7 ± 10.2		2.8 ± 1.9	
60–64	184 (31)	9.1 ± 9.4		11.5 ± 10.9		3.0 ± 1.9	
65–69	138 (24)	12.8 ± 10.4		13.0 ± 12.3		3.0 ± 1.6	
70–74	40 (7)	14.0 ± 12.8		18.5 ± 15.8		3.1 ± 2.2	

Education							
Registered nurse diploma	340 (58)	11.0 ± 9.9	0.2	13.6 ± 11.1	0.05	3.0 ± 2.0	0.3
Bachelor’s degree	160 (27)	10.4 ± 8.9		10.8 ± 12.4		2.7 ± 1.6	
Master’s or doctorate degree	87 (15)	9.0 ± 10.5		12.4 ± 11.9		2.9 ± 1.9	

Husband’s education							
High school or less	137 (23)	12.2 ± 10.5	0.06	15.1 ± 13.2	0.01	3.2 ± 2.3	0.06
College education	313 (53)	9.9 ± 9.9		12.2 ± 11.5		2.8 ± 1.7	
No longer married or unknown	137 (23)	10.4 ± 8.5		11.3 ± 9.8		2.9 ± 1.7	

Smoking status							
Never	234 (40)	9.8 ± 9.2	0.3	11.3 ± 10.8	0.07	2.7 ± 1.8	0.01
Past	308 (52)	10.9 ± 10.2		13.6 ± 12.4		3.0 ± 2.0	
Current	45 (8)	11.6 ± 9.4		12.9 ± 9.2		3.6 ± 1.6	

Alcohol consumption, long-term mean (g/day)							
< 1	141 (24)	9.4 ± 9.1	0.3	12.2 ± 11.2	0.6	2.5 ± 1.5	0.01
1–4	200 (34)	10.6 ± 9.9		12.4 ± 11.5		2.9 ± 2.0	
5–14	155 (26)	10.9 ± 9.8		13.1 ± 13.1		3.1 ± 2.1	
≥ 15	69 (12)	12.2 ± 10.6		14.2 ± 10.3		3.4 ± 1.7	

Energy expended on regular physical activity (MET-hr/week)							
< 16	284 (48)	11.0 ± 8.8	0.2	12.3 ± 11.2	0.5	2.9 ± 1.8	0.6
≥ 16	300 (51)	10.0 ± 10.4		12.9 ± 12.1		2.9 ± 2.0	

Postmenopausal hormone use							
Premenopausal	44 (7)	10.3 ± 8.2		13.5 ± 11.8		2.3 ± 1.5	
Postmenopausal
Never used	140 (24)	11.6 ± 10.1	0.5	12.5 ± 12.5	0.2	3.6 ± 2.3	< 0.0001
Used in the past	222 (38)	9.9 ± 10.9		11.4 ± 12.1		3.1 ± 1.8	
Currently use	181 (31)	10.5 ± 8.2		14.0 ± 10.1		2.2 ± 1.4	

Aspirin use (times/week)							
< 3	460 (78)	9.9 ± 9.3	0.02	11.8 ± 11.0	0.001	2.9 ± 1.8	0.1
≥ 3	74 (13)	12.7 ± 12.0		16.5 ± 14.8		3.2 ± 2.6	

Ibuprofen use							
No	435 (74)	10.4 ± 10.0	0.6	12.5 ± 11.4	0.7	2.9 ± 1.9	0.8
Yes	152 (26)	10.9 ± 8.9		13.0 ± 12.3		2.9 ± 2.0	

Vitamin E use							
No	322 (55)	10.9 ± 9.9	0.4	13.4 ± 12.3	0.2	3.0 ± 2.0	0.1
Yes	215 (37)	10.1 ± 10.0		11.9 ± 11.1		2.8 ± 1.7	

TICS score < 31							
No	565 (96)	10.3 ± 9.5	0.006	12.3 ± 11.4	0.001	2.9 ± 1.8	0.02
Yes	22 (4)	16.1 ± 13.3		20.4 ± 14.7		3.8 ± 2.7	

MET-hr, metabolic equivalent-hours.

a *p*-Values for each trait and lead biomarker correspond to the *F* test of the overall association between the trait and the lead biomarker.

b Women with bone lead assessments (total *n* = 587); denominators for patella lead, blood lead, alcohol intake, physical activity, aspirin use, and vitamin E use are slightly smaller.

**Table 2:** Adjusted^a^ mean difference (95% CI) in overall standardized cognitive test score per SD increment in lead biomarker.

	All cognitive tests		All cognitive tests except letter fluency	
	Mean difference (95% CI)	*p*-Value	Mean difference (95% CI)	*p*-Value
Per SD increment in lead biomarker				
Tibia bone lead (SD, 10 μg/g)	−0.040 (−0.085 to 0.004)	0.08	−0.051 (−0.099 to −0.003)	0.04
Patella bone lead (SD, 12 μg/g)	−0.012 (−0.056 to 0.033)	0.61	−0.033 (−0.080 to 0.014)	0.17
Blood lead (SD, 2 μg/dL)	−0.015 (−0.069 to 0.039)	0.59	−0.016 (−0.071 to 0.039)	0.57
Per year in age at cognitive assessment^b^	−0.018 (−0.029 to −0.008)	0.003	−0.017 (−0.028 to −0.007)	0.002

a Based on GEE models adjusted for age and age-squared at lead assessment, age at cognitive assessment, education, husband’s education, alcohol consumption, smoking status, physical activity, aspirin use, ibuprofen use, use of vitamin E supplements, menopausal status and postmenopausal hormone use, lead substudy source, and cognitive substudy source.

b Adjusted for education, husband’s education, lead substudy source, and cognitive substudy source.
