# Erratum

**Published:** 2009-06

In “Cumulative Exposure to Lead in Relation to Cognitive Function in Older Women” [Environ Health Perspect 117:574–580 (2009)], Mark Weisskopf’s middle initial is incorrect. Instead of “Marc A. Weisskopf,” it should be “Marc G. Weisskopf.”

The authors apologize for the error.

